# Advanced Neuromonitoring Modalities on the Horizon: Detection and Management of Acute Brain Injury in Children

**DOI:** 10.1007/s12028-023-01690-9

**Published:** 2023-03-22

**Authors:** Tiffany S. Ko, Eva Catennacio, Samuel S. Shin, Joseph Stern, Shavonne L. Massey, Todd J. Kilbaugh, Misun Hwang

**Affiliations:** 1 Department of Anesthesiology and Critical Care, Children’s Hospital of Philadelphia, Philadelphia, USA; 2 Division of Neurology, Department of Pediatrics, Children’s Hospital of Philadelphia, Philadelphia, USA; 3 Department of Neurosurgery, Hospital of the University of Pennsylvania, Philadelphia, USA; 4 Department of Radiology, Children’s Hospital of Philadelphia, University of Pennsylvania, Philadelphia, USA

**Keywords:** Pediatric ICU, Pediatric emergency medicine, Acute brain injuries, Neuroimaging, Neurophysiological monitoring, Hypoxia–ischemia, Brain, Diagnostic ultrasound

## Abstract

Timely detection and monitoring of acute brain injury in children is essential to mitigate causes of injury and prevent secondary insults. Increasing survival in critically ill children has emphasized the importance of neuroprotective management strategies for long-term quality of life. In emergent and critical care settings, traditional neuroimaging modalities, such as computed tomography and magnetic resonance imaging (MRI), remain frontline diagnostic techniques to detect acute brain injury. Although detection of structural and anatomical abnormalities remains crucial, advanced MRI sequences assessing functional alterations in cerebral physiology provide unique diagnostic utility. Head ultrasound has emerged as a portable neuroimaging modality for point-of-care diagnosis via assessments of anatomical and perfusion abnormalities. Application of electroencephalography and near-infrared spectroscopy provides the opportunity for real-time detection and goal-directed management of neurological abnormalities at the bedside. In this review, we describe recent technological advancements in these neurodiagnostic modalities and elaborate on their current and potential utility in the detection and management of acute brain injury.

## Introduction

Over the last decade, management-based improvements in survival of pediatric critical care patients have led to a shift in focus beyond survival to neuroprotection and quality of life. Primary neurologic diagnoses requiring critical care intervention occur in 26.9 per 100,000 US children per year [1]. Seizure disorders and traumatic brain injury (TBI) are the most common primary neurological diagnoses [1–3], accounting for more than 50% of all pediatric neurocritical care admissions. Neurological infection, hydrocephalus, and stroke are also common primary neurologic admissions. TBI remains a leading cause of death and disability in children in the United States [4]. In addition to direct brain injury, cardiac or respiratory insufficiency from congenital disease or exogenous insults (e.g., infection, aspiration) may also result in secondary hypoxic–ischemic encephalopathy (HIE). Early detection of neurological vulnerability paired with targeted brain-directed management strategies is essential to mitigating the lifelong impact of pediatric brain injury.

In the event of suspected moderate or severe closed head injury in children, noncontrast head computed tomography (CT) is widely accepted as the frontline diagnostic modality to determine the presence of life-threatening intracranial injuries and skull fractures [5–8]. The development of portable CT has also extended access to critically ill patients where transport is not feasible [9, 10]. However, CT imaging falls short in detection of parenchymal contusions, diffuse axonal injury, nonhemorrhagic intracranial hypertension, and perfusion abnormalities [11, 12]. In fact, frontline CT imaging may fail to detect acute ischemic stroke in as many as 47% of children [13, 14].

Due to these limitations and evidence of the elevated cancer burden in children associated with ionizing radiation exposure [15, 16], there has been increasing adoption of frontline magnetic resonance imaging (MRI) [8, 17–22] and head ultrasound (HUS) [23, 24, 25, 26, 27]. However, diagnostic MRI and HUS is only triggered following presentation of injury and is limited to snapshots over time due to equipment and staffing requirements. Delayed detection increases injury severity and, in the critical care setting, limits opportunities for optimization of management at the bedside. Continuous noninvasive neuromonitoring modalities, including electroencephalography (EEG) and near-infrared spectroscopy (NIRS), are poised to fill this critical gap to enable more timely detection, quantification, and treatment of neurological abnormalities [28–30].

In this review, we provide a technical summary of both existing and emerging MRI, HUS, EEG, and NIRS techniques and elaborate on their current and potential indications in the detection and management of acute brain injury in pediatric populations (Table 1).

**Table 1:** Neuromonitoring modalities for acute brain injury: limitations and emerging advancements

	Clinical information	Anatomic	Staffed	Portable	Continuous	Other limitations	Advancements
MRI	Whole-brain localization of soft tissue	X	X			Cost Patient transport	Rapid sequences Perfusion Metabolism Tractography
Ultrasound	Anatomical imaging of soft tissue Perfusion	X	X	X		Field-of-view Operator variability	Low-flow sensitivity Microvascular perfusion Elastography Intracranial pressure
EEG	Seizure detection Sedation		X	X	X	Electrical interference Interpretation expertise	Artifact rejection Quantitative metrics Localization
Optical	Cerebral oxygen saturation			X	X	Intersubject variability	Perfusion Oxygen metabolism Intracranial pressure Cerebral autoregulation

## Advances in Neuromonitoring

### Magnetic Resonance Imaging (MRI)

In the emergent and critical care setting, rapid MRI sequences with reduced duration on the order of seconds for individual sequences and < 30-min total scan time [18, 31] has made nonsedated pediatric imaging more successful and likely underlies increased utilization in acute brain injury [17, 25, 27]. Faster acquisition techniques are increasingly being explored, in addition to the recent emergence and utilization of portable MRI scanners [32]. Brain MRI now serves as the reference standard for detection of brain injury, including stroke [14, 21, 33], status epilepticus [34], and TBI [35]. The sections below detail key advanced MRI techniques that have provided value in the diagnosis and prognostication of pediatric brain injury in the critical care setting.

#### Arterial Spin Labeling

Arterial spin labeling (ASL) enables noninvasive imaging of cerebral blood flow (CBF) on a voxel-by-voxel basis by detecting the arrival of magnetically tagged water molecules in blood [36, 37]. These water molecules are tagged by an inversion pulse as they transit through a cross-sectional tagging plane localized to the carotid and vertebral arteries in the neck. If the T1 relaxation time of arterial blood is either assumed or individually measured, absolute blood flow units of mL/100-g tissue/minute may be estimated using the flow-modified Bloch equation solution. A single inversion pulse tag results in ~ 1–2% change in signal from baseline [38]; enhancing this small signal contrast is an ongoing focus of ASL optimization.

The ability to noninvasively localize and quantify perfusion alterations provides critical physiologic information that may be used to detect acute neurological insults or monitor efficacy of therapeutic treatment. ASL has seen increasing routine adoption in adult patients with suspicion of stroke [39] and with status epilepticus [40, 41]; application in pediatric populations remains an active area of study due to the significant impact of brain maturation on optimal sequence parameters and on the accuracy of CBF quantification [42–45]. ASL can currently provide relative information regarding perfusion across different brain regions; however, it cannot provide absolute values for CBF that could be used to classify hypoperfused, ischemic, and infarcted brain tissue.

When ASL is combined with a diffusion-weighted imaging (DWI) acquisition, a mismatch showing high diffusivity on DWI with low blood flow on ASL may help identify at-risk hypoperfused regions prior to infarct [39, 46]. ASL measurements of CBF have also demonstrated correlation with brain tumor severity grading in children [47, 48] and decreased perfusion in children with uncompensated hydrocephalus that is reversed following neurosurgical intervention [49, 50]. Evidence of the utility of ASL for seizure localization and prognostication of refractory status epilepticus in children is also emerging [51–59]. Perfusion abnormalities are observed more commonly in focal versus generalized seizures [55, 58]. Lam et al. [57] found presurgical ASL abnormalities had 100% sensitivity but 23% specificity for predicting positive histopathology results in 11 surgical patients with focal epilepsy. Further study is needed to clarify the presentation of hyperperfusion versus hypoperfusion abnormalities as a function of postictal timing, pathogenesis, and patient age.

#### Diffusion Tensor Imaging

Diffusion tensor imaging (DTI) is an advanced computational derivative of DWI that permits characterization of white matter structure and function [60]. Commonly reported voxel-specific DTI metrics are the fractional anisotropy (FA), reflecting the degree of directed diffusion, and the mean diffusivity (MD), the average magnitude of diffusion in all directions. With axonal injury, FA is commonly reduced and MD is elevated. Computational methods may be used to connect neighboring voxels with similar directed diffusion to reconstruct axonal fiber tracts. Advanced analysis of DTI data includes tract-based spatial statistics, tractography, and functional connectivity via application of graph theory [61].

DTI sensitivity to microstructural abnormalities and disruptions of white matter fiber tracts has shown significant correlation with functional status, even in the absence of apparent abnormalities on conventional T1 and T2 structural imaging. In neonates with HIE, severity of DTI abnormalities, present in white matter, basal ganglia, posterior limb of the internal capsule, and watershed areas, was correlated with seizure severity [62]. Significant reductions in FA observed at 3 months post injury persist up to 18 months following moderate to severe pediatric TBI, which may underlie functional deficits [63]. DTI parameters have also shown good correlation with plasma biomarkers of injury, such as neurofilament-light and glial fibrillary acidic protein, in both clinical [64] and animal models of TBI [65]. In children with status epilepticus, DTI injury severity grade was also correlated with elevated serum S100B protein levels, a marker of brain injury [66]. Further study is necessary to determine whether DTI abnormalities may in fact precede seizure burden or result secondary to injury.

**Fig. 1:**
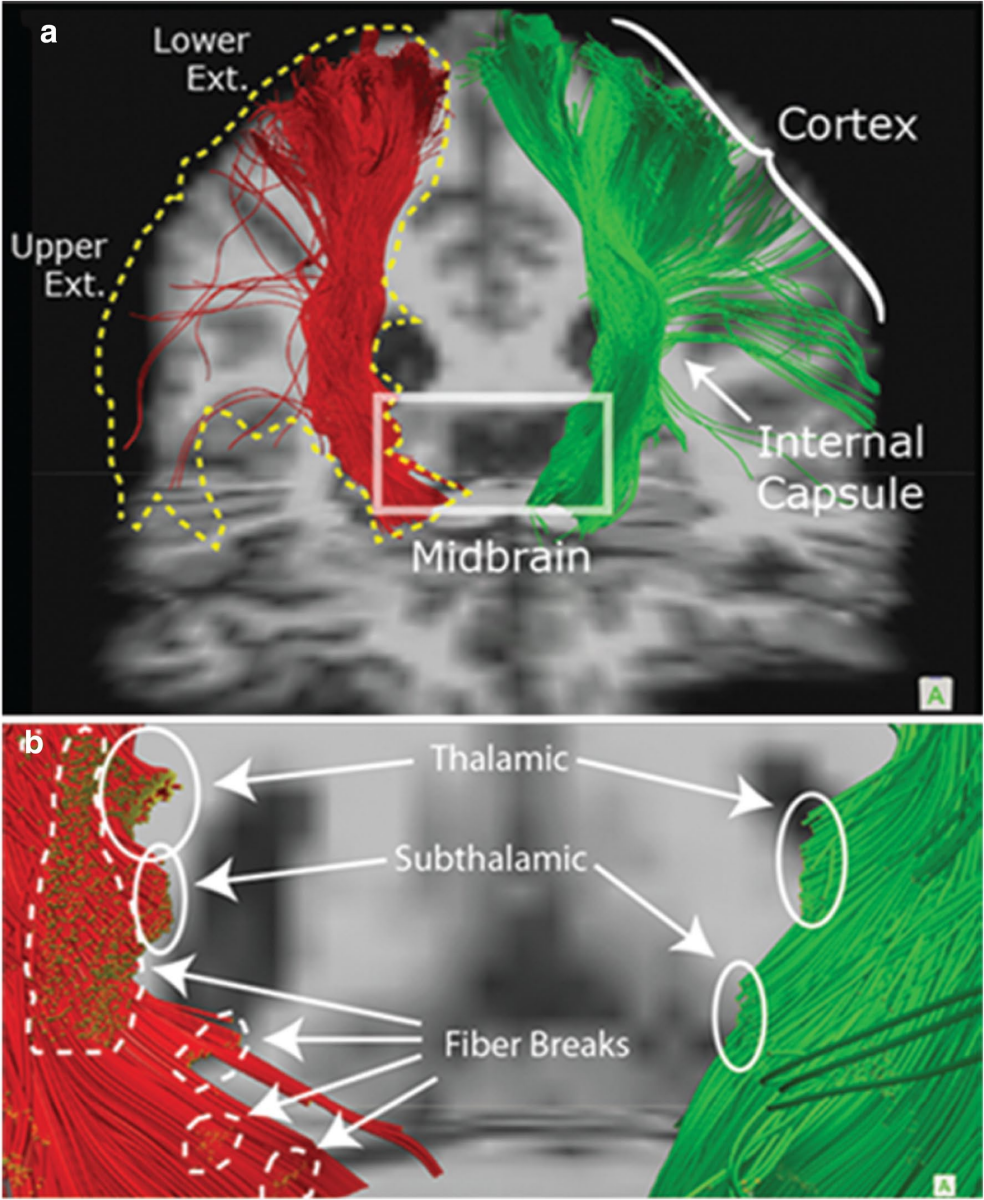
Fiber tractography using diffusion imaging in a case of TBI. In this patient with severe TBI, high-definition fiber tractography showed details of white matter injury such as local disconnection in the subcortical regions (white circles) and well as gross disconnections of large regions (yellow-dotted outline). Abbreviations: TBI, traumatic brain injury. (Figure adapted from Shin et al. (2012) with permission. JNS Publishing Group holds the copyright to this figure, and subsequent use of this material requires permission from JNS Publishing Group.)

Challenges remain in improving the accuracy of tract reconstruction. DTI reconstruction based on increased directional density (250 + directions), termed high-definition fiber tractography, is capable of resolving complex fiber crossings using diffusion spectrum imaging and has demonstrated increased fidelity and robustness of tract reconstruction [67, 68]. Specifically, detailed information of white matter microstructural injury can be visualized and quantified (Fig. 1). However, further characterization is needed for pediatric brain injury given that the degree of myelination differs from adults. Because TBI pathophysiology has significant differences between pediatric and adult population, fiber tractography studies comparing the two are needed in the future.

#### Magnetic Resonance Spectroscopy and Imaging

Magnetic resonance spectroscopy (MRS) provides noninvasive concentration profiling of several significant metabolites, including small molecular weight amino acids, carbohydrates, fatty acids, and lipids, to aid in the detection and treatment of brain tumors, infection of the central nervous system, mild TBI, HIE, and other subclinical acute insults (Table 2) [69–73]. Although conventional MRI focuses on imaging the proton of the hydrogen molecule (^1^H) in water, MRS utilizes the magnetic field signal of hydrogen protons on these other molecular species to quantify alterations in metabolism, neuronal cell death, and demyelination.

**Table 2:** Magnetic resonance spectroscopy: metabolites and diagnostic utility

Metabolite	Physiology	Diagnostic utility
Cho	Precursor component of cell membranes and myelin	(+) cellular proliferation; viral infection; brain tumors (−) hypothermia
Cr	Metabolic product of creatine phosphate breakdown during protein metabolism	(+) metabolic activity; energetic reserve; inflammation (−) pilocytic astrocytoma
Gln	Precursor for neuronal synthesis of glutamate	(+) acute hypoxic-ischemia; brain tumors
Glu	Excitatory neurotransmitter	(+) brain maturation; epileptic tissue; excitotoxicity
Lac	Metabolic product of anaerobic glycolysis	( +) acute hypoxic-ischemia; brain tumors
Lip	Fatty acid component of cell membranes	(+) apoptosis/necrosis
mIns	Glial metabolite	(+) gliosis; astrocytoma (−) brain maturation
NAA	Neuronal metabolite	(+) neuronal integrity; brain maturation (−) acute hypoxic-ischemia; apoptosis/necrosis; acute seizure
Suc	TCA cycle intermediate metabolite	(+) infection

*Abbreviations:* *Cho*, Choline; *Cr,* Creatinine; *Gln,* Glutamine; *Glu,* Glutamate; *Lac,* Lactate; Lip, CH_3_ (methyl) and CH_2_ (methylene) group lipid molecules; mIns, myo-inositol, *NAA,* N-acetylaspartate; *Suc,* Succinate; TCA, tricarboxylic acid ; (+) increase in metabolite; (−) decrease in metabolite

MRS yields critical diagnostic information in neonates with perinatal asphyxia in which N-acetylaspartate (NAA) concentration, the lactate (Lac)/NAA ratio, and the Lac/choline ratio have been correlated with poor outcome [74–78]. Recent results of the MARBLE trial confirm the high predictive utility of reduced thalamic NAA concentration (AUC = 0.99) and elevated Lac/NAA ratio > 0.22 (AUC = 0.94), measured within 4–14 days after birth, for adverse neurodevelopmental outcomes at 2 years of age [79]. Significant reduction in MD and elevation of the Lac/NAA ratio are observed within the first 2 days after birth despite unremarkable findings on T1 and T2 [76]. The Lac/NAA ratio was most highly associated with outcomes at 18–22 months of age when acquired within 24–96 h of life versus 7–14 days [80].

In TBI, NAA level has been demonstrated as a measure of neuronal and axonal integrity, with reduction in the NAA level following TBI [81, 82]. An additional parameter that is widely used includes choline, which is considered a biomarker of cell membrane turnover [83]. Given the variability of NAA levels at different time points after TBI, as well as the study participant’s age, normalization, such as using the NAA/creatinine ratio or comparison to an appropriately aged and post-TBI time-matched control group, is important in MRS research. MRS has also demonstrated prognostic utility in TBI with severity of NAA decline and choline and Lac increase correlated with injury severity in the acute and subacute injury period [11, 84–87]. Early detection of injury severity and characterization of associated metabolic derangements may aid in the development of neuroprotective strategies to mitigate secondary injury.

#### Contrast-Enhanced MRI Techniques

Contrast-enhanced MRI techniques provide high-resolution anatomical and structural information to improve diagnostic discrimination of intracranial vascular malformations, vasculitis, and brain tumors in children [88, 89]. Intravenously administered contrast agents are most commonly paramagnetic gadolinium ion complexes, which shorten relaxation times of surrounding water protons. These complexes permeate the blood–brain barrier and create positive contrast enhancements (hyperintensities) in surrounding endothelial and extravascular tissue. Within the last 5 years, several gadolinium contrast agents have received regulatory approval for use in children of all age groups [89–91]. The potential risks of gadolinium contrast administration may be offset by improved diagnostic accuracy [92–94], in particular in pediatric stroke when time-to-diagnosis is vital for the prevention of neurological injury and death, and by improved prognostication [95–97]. Alternative contrast agents, including liposomes, micelles, and inorganic nanoparticles (e.g., ferumoxytol), remain an active area of study [98, 99].

“Black-blood” T1-weighted vessel wall imaging (VW-MRI) permits contrast-enhanced visualization of periarterial vessel wall structure in the anterior circulation [100–102] and has been increasingly utilized in pediatric stroke [103]. Although a degree of linear enhancement along medium- to large-sized arteries is normal in children [104], atypical enhancement in arteries bordered by cerebrospinal fluid (CSF) and/or hemispheric asymmetry provides localized discrimination of arteriopathies [96, 105–108], which contribute to as many as 50% of pediatric arterial ischemic strokes (AIS) and are associated with higher stroke recurrence and poor outcomes [109]. In a retrospective study of 16 study participants with pediatric AIS, strong vessel wall enhancement, defined on a 3-point scale (none, mild, strong) [110], at stroke presentation was correlated with progressive arteriopathy in 83% of cases [96]. VW-MRI may also improve recognition of primary vasculitis/angiitis of the central nervous system [111, 112], which has been observed in 24% of pediatric AIS cases [113] but is likely underestimated. Continued study is needed to understand the age dependence of vessel wall enhancement and if magnitude of enhancement may be a biomarker of inflammatory disease progression.

### Ultrasound

HUS is an important neuroimaging modality in the pediatric neurocritical care setting due to its portability, safety, and ability to capture real-time bedside images. In neonates and infants, the open fontanelles serve as a natural acoustic window. In children with closed fontanelles, the thin squamous portion of the temporal bone can be used as the acoustic window. Despite the availability of advanced neuroimaging techniques, HUS, due to its unique advantages, remains a pillar of brain imaging in pediatric neurocritical care.

#### Technical Considerations

Ultrasound frequency waves in the MHz range are generated and transmitted into tissue by a piezoelectric transducer; the echoes reflected from the tissues are also detected by the transducer. The echoes contain spatial and contrast information, which generates electrical signals and can be quantified and extrapolated into images of internal tissues and organs. Conventional HUS techniques used include grayscale, B-mode HUS, and transcranial Doppler (TCD).

Selective filtering of returning echoes allows the visualization of information beyond that of conventional grayscale ultrasound. In this regard, Doppler ultrasound assesses frequency changes of returning echoes and can determine whether there is movement of tissue toward or away from the transducer. The technique allows assessment of blood flow through macrovessels and interferences about tissue health [114, 115]. Spectral Doppler offers quantitative flow characteristics, such as velocity, resistive index, and pulsatility. To date, TCD ultrasound has served an important role in screening for patients with at-risk ischemia, ischemia, and/or vasospasm. Namely, TCD serves as the standard of care screening tool for patients with sickle cell [116, 117]. Major limitations of Doppler ultrasound technology include high sensitivity to motion and insensitivity to slow flow, such as that of microvessels. Emerging advanced ultrasound techniques are being studied to overcome the inherit limitations of conventional techniques.

#### Emerging Techniques

##### Microvascular Imaging

**Fig. 2:**
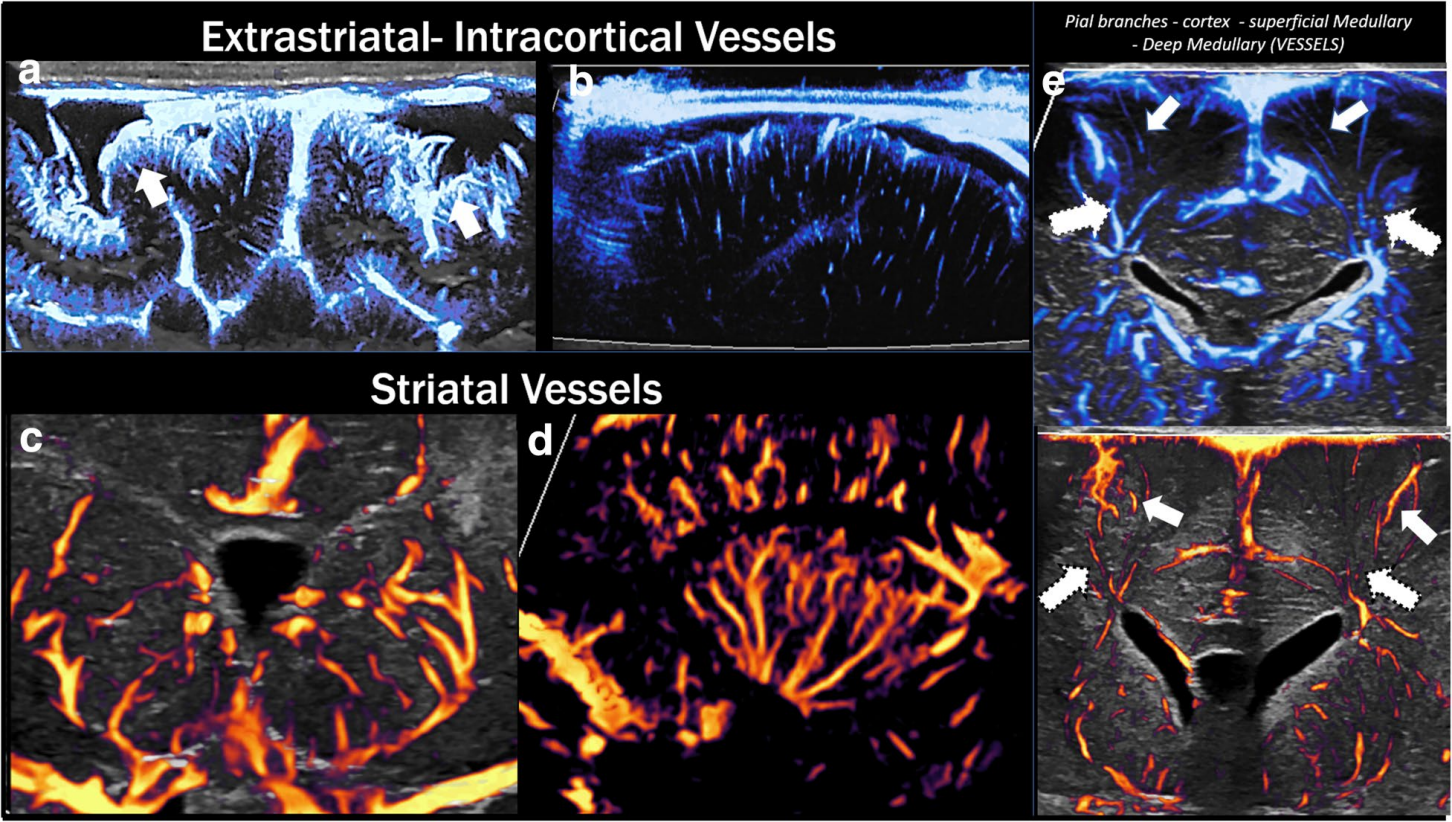
Microvascular architecture of the brain. Microvascular imaging (MVI) anatomic features: superficial cortical vessels and pial branches (arrows) (**a**), microvessels going through the deeper cortical layers (**b**), striatal vessels in the coronal and sagittal plane (**c**, **d**), and superficial (arrows) and deep (dotted arrows) medullary vessels (**e**)

Microvascular imaging (MVI) is an advanced Doppler technique that permits visualization of slow flow in cerebral microvessels (Fig. 2). In MVI, an advanced adaptive wall filter is used to suppress tissue clutter or static noise while detecting low velocity flow (< 2.5 cm/s) [118, 119, 120, 121]. Two modes of MVI are available: monochrome MVI and color MVI. Monochrome MVI highlights flow in microvessels in the dark background with the grayscale ultrasound subtracted. Color MVI allows visualization of microvessels overlaid on the grayscale anatomical background.

There is emerging evidence that MVI may be used to detect cerebral microvessels in the brain, thereby attaining information on normal neurodevelopment and functional alterations related to a variety of brain pathologies [122]. Initial studies have reported on feasibility of visualizing striatal and nonstriatal (superficial and deep cortical) microvessels using this technique [119, 120]. Age-dependent differences in the morphology of cerebral microvessels can be observed with lesser visualization and maturity of both the striatal and nonstriatal vessels in the brains of preterm versus term infants [119]. In hemispheric stroke, acute hypoperfusion or reperfusion response to initial ischemic insult can be detected, especially when the abnormality is asymmetric and/or focal in distribution [122, 123]. Real-time bedside detection could allow timely individualized management. Although MVI has predominantly been applied in infants, application in children with closed fontanelles is plausible using the temporal bone as the acoustic window.

##### Contrast-Enhanced Ultrasound

**Fig. 3:**
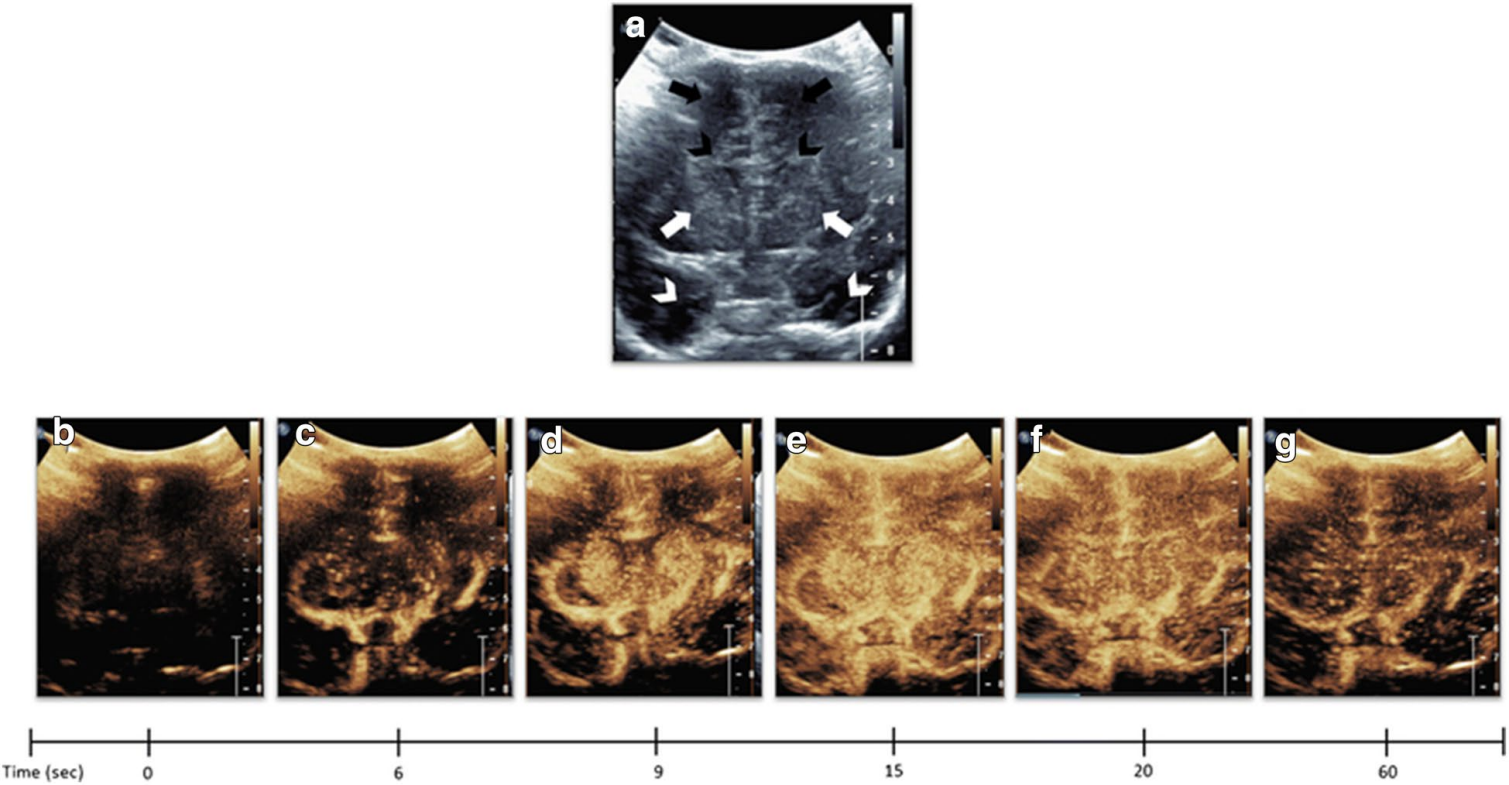
Dynamic microbubble wash-in on midcoronal brain scan in a normal study participant. **a** Denotes a midcoronal grayscale ultrasound scan through the brain of a normal study participant showing bilateral frontal lobes (orange arrows), frontal horns (yellow arrowheads), basal ganglia (red arrows), and temporal lobes (white arrows). **b**–**g**, Demonstrates dynamic microbubble wash-in through the midcoronal slice through the brain on a contrast specific mode from the time of injection (time 0) to 1 min. Note that the microbubbles flow into the partially visualized Circle of Willis by 13 s. By 15 s, relatively more avid enhancement to the basal ganglia with respect to the remainder of the brain is seen. Further enhancement of the cortex but with relative hyperenhancement of the basal ganglia is noted at 20 s. Wash-out of contrast from both the basal ganglia and cortex begins at 25 s and further wash-out noted at 60 s. Note that Fig. 2 images were obtained with EPIQ scanner (Phillips Healthcare, Bothell, WA) and C5–1 transducer with settings of 12 Hz and MI of 0.06. (Fig. reproduced from Hwang (2019) [270] with permission.)

Contrast-enhanced ultrasound (CEUS) is a technique that uses the intravenous injection of an ultrasound contrast agent called microbubbles for assessment of tissue perfusion (Fig. 3). These contrast agents can be given in bolus injections or continuous infusion, though the former is the more widely utilized method in the clinical setting. Microbubbles oscillate on insonation and emit echoes that are detected by the transducer to create signal. Microbubbles are approximately 2–3 µm in size, about half the size of red blood cells, thereby coursing through the capillaries of organs. Commercially utilized microbubbles contain biologically inert gas in the core and phospholipid monolayer in the shell. If injected into the body, the gas is cleared through the lungs. CEUS allows spatiotemporal assessment of tissue perfusion in real time, complementing conventional ultrasound, without the need for transportation, sedation, or radiation. Dynamic perfusion kinetics are quantified using the standardized time-intensity curve, wherein signal intensity in a region of interest is quantified over time [124]. CEUS uses a low mechanical index, typically less than 0.2, to avoid microbubble destruction. Use of a curved transducer between 2 and 8 MHz allows for optimal visualization, as this frequency coincides with the resonant frequency of the microbubbles.

Although the use of CEUS in the brain is relatively new, there is great potential for quantitative and qualitative evaluation of many neurological pathologies and evaluation of cerebral perfusion for diagnostic and prognostic use. Given the inherent challenges of detecting symmetric and/or diffuse pattern injury as can be seen in hypoxic–ischemic injury, Hwang et al. assessed the feasibility of quantitative detection by evaluating the ratio of basal ganglia to cortex perfusion [125]. In stroke, hyperacute hypoperfusion or reperfusion response can be assessed. In fact, much of the CEUS studies in stroke have been in children and adults with closed fontanelles [124, 126]. In using the temporal bone as the acoustic window, it is important to recognize that the acoustic-bone impedance can lead to decreased signal on the contralateral side [127]. If using the bolus injection technique, two bolus injections and interrogation of hemispheric temporal bone each are therefore desirable so as not to attribute apparent signal reduction in the contralateral brain to injury.

**Fig. 4:**
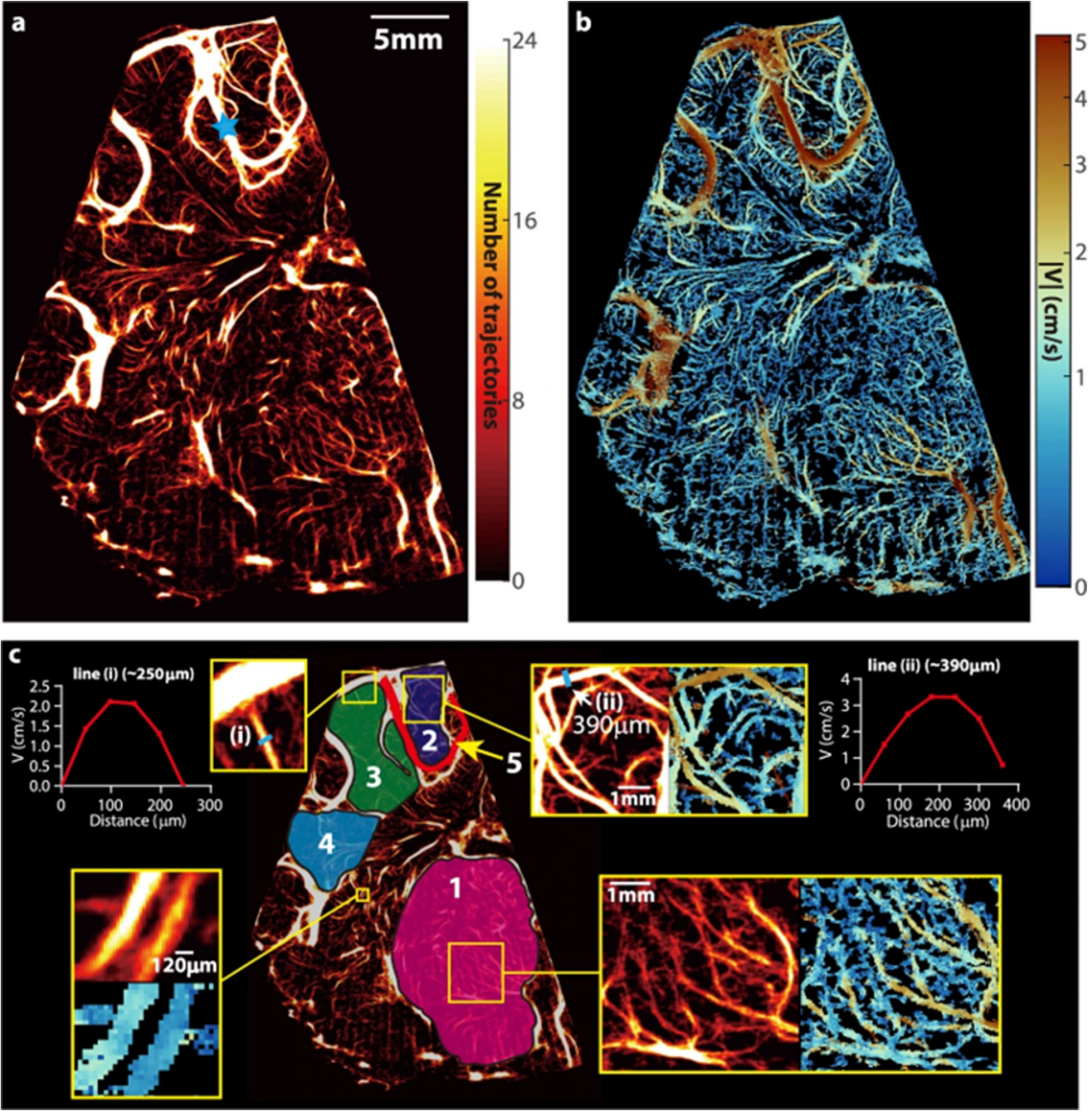
Cerebral vascular map and velocity distribution for a piglet at baseline intracranial pressure. **a**, A heatmap of all the trajectories containing at least four exposures visualizing the micro- and macro-vascular distributions in a coronal plane. The blue star marks the location of pulsed-wave Doppler ultrasound measurement. **b**, The corresponding time-averaged velocity distribution. **c**, Several sub-regions are labeled for statistical analysis of perfusion, including the micro-vessels in the thalamus (#1), several parts of the cortex (#2, #3, and #4), as well as a macro blood vessel (#5). A few regions are magnified to provide a closer view of the current regions of interest. Sample velocity profiles across two different blood vessels are also provided in (**c**). One measurement is made for this sample case, where 5760 images are used to generate results. (Figure reproduced from Zhang et al. [128] under Creative Commons license—http://creativecommons.org/licenses/by/4.0/.)

A recent article by Zhang et al. has shown that cerebral microvascular flow as assessed using microbubbles can provide robust measures of intracranial pressure (ICP) and brain ischemia in hydrocephalus [128]. In this study, advanced particle tracking is applied in a neonatal porcine hydrocephalus model to track individual microbubble across thousands of ultrasound frames to derive cerebral microvascular morphology and velocity (Fig. 4). A strong correlation between cerebral microvascular velocity and ICP was observed, with incremental reduction in flow velocity with increasing pressure. Interestingly, the onset of brain ischemia coincided with drastic reduction in cortical flow. As such, spatial changes in cerebral microvascular flow can serve as a valuable biomarker of disease and help guide therapy in the future if further validated. Beyond hydrocephalus, the potential utility of CEUS has been described in a wide range of neurologic diseases, including brain tumors and vascular malformations [124].

##### Elastography

Elastography is an advanced ultrasound technique that allows for the noninvasive assessment of brain tissue stiffness [129, 130, 131]. Brain tissue stiffness can change with many factors, including myelination, edema, and ICP. There are two primary ultrasound elastography methods used: strain-based and shear-wave-based elastography. It should be noted that strain elastography provides semiquantitative measures of brain stiffness whereas shear wave elastography provides quantitative measures of brain stiffness, such that the latter is most widely used in brain applications. In strain-based elastography, external pressure is applied by the operator, and Young’s modulus is calculated from tissue displacement from the applied external pressure. Stiffer lesions deform less to external pressure and therefore have higher Young’s modulus values. In shear-wave-based elastography, high-intensity acoustic waves induce perpendicularly propagating shear waves from tissue, which are then captured by the ultrasound probe to measure shear wave velocity (SWV). Young’s modulus is estimated from SWV; greater SWV signifies greater tissue stiffness and a greater Young’s modulus value.

**Fig. 5:**
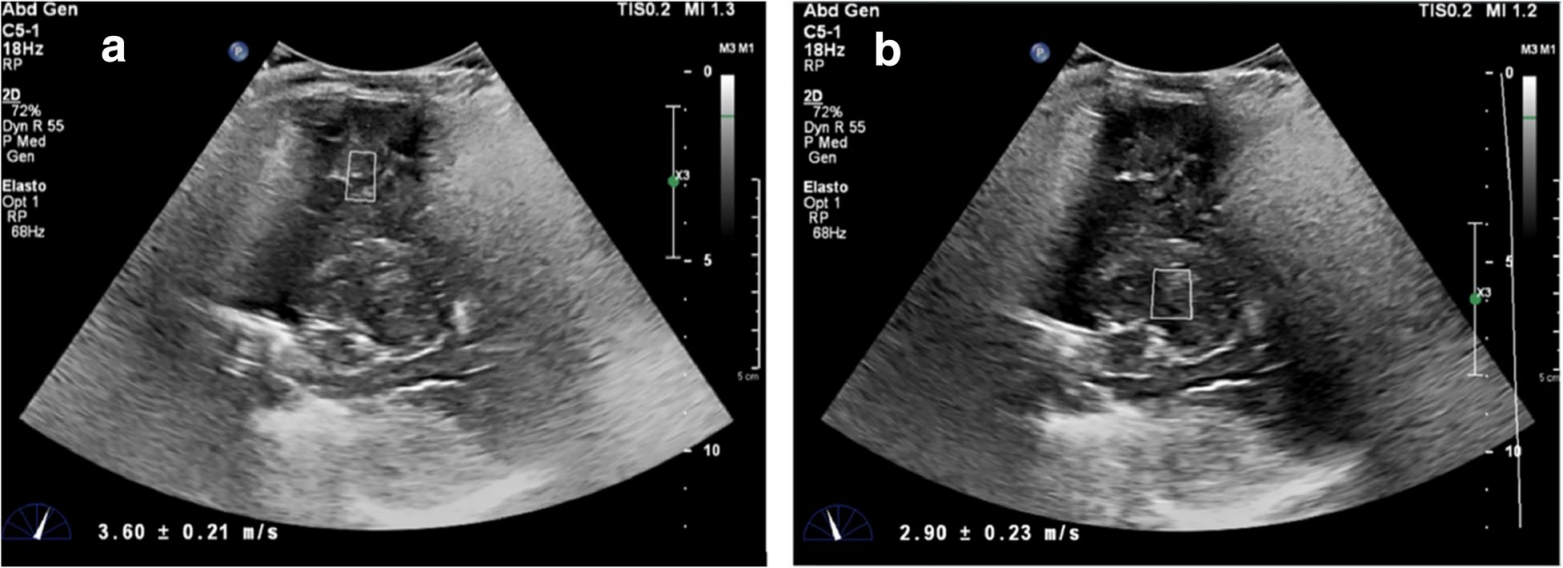
Ultrasound elastography of an infant with severe anoxia. This is a 6-month-old, former 37-week gestational infant with unwitnessed cardiac arrest at home. Capillary gas pH 6.8, pCO2 43, pO2 144, and bicarbonate 3.5. End tidal CO_2_ during resuscitation were in the 20 s and high 40 s. He was imaged 30 min after return of spontaneous circulation. Values were elevated in the gray and white matter 3.6 ± 0.21 m/s (**a**) and basal ganglia 2.9 ± 0.23 m/s (**b**). (Reproduced from deCampo and Hwang [130] with permission.)

Brain elastography has been applied predominantly in infants due to open acoustic fontanelles. Age- and region-dependent differences in brain elasticity have been shown [132–136]. Applications in disease diagnostics remain sparse but are emerging; it has been shown that brain stiffness increases in a focal epileptic zone [137], can either increase or decrease depending on the timing of hypoxic–ischemic injury, and increases with raised ICP [138]. In situations when high ICP is suspected, elastography may serve as a useful adjunct tool (Fig. 5).

### Electroencephalography (EEG)

EEG, initially developed in the 1920s [139], has traditionally been used for the diagnosis and management of epilepsy, one of the most common chronic neurologic illnesses in children [140]. However, due to exquisite temporal resolution and advances in real-time interpretation, continuous EEG (cEEG) is increasingly being utilized as a neuromonitoring tool in intensive care units for a variety of indications in both neonatal [141, 142] and pediatric/adult populations [143, 144]. The goals of monitoring are to (1) identify background patterns and features that may indicate underlying cerebral dysfunction, direct toward specific etiologies, or provide prognostic data and (2) detect and quantify electrographic seizures and assess response to treatment. It is well established that there is a large burden of EEG-only (subclinical) seizures in critically ill populations that require cEEG monitoring for detection. Review of EEG activity can identify patterns suggestive of seizures, structural abnormalities (e.g., slowing of cerebral activity in a focal region), diffuse cerebral dysfunction, or specific disease processes (e.g., extreme delta brush patterns seen in autoimmune encephalitis).

#### Technical Considerations

During an EEG recording, an array of electrodes is placed on the scalp (using a conductive paste and/or glue) in standardized locations (10–20 system of electrode placement); the number of electrodes used may vary by patient age and head circumference, with a sparser array used in neonates [145]. The electrical activity detected by an EEG electrode represents the summation of excitatory or inhibitory postsynaptic potentials in groups of underlying cortical pyramidal neurons firing simultaneously [146]. This generates an electrical field that diminishes in strength with increasing distance from the source of the electrical potential. Differential amplification of electrical potentials between pairs of inputs (either two electrodes or an electrode and a reference signal) are compared. Combinations of electrodes, called montages, aid in localizing where electrical field potentials are maximal. Electrical waveforms themselves can be characterized by their frequency, voltage, morphology, and rhythmicity and interpreted as normal or abnormal in the context of age, behavioral state (awake, asleep, indeterminate), and other factors, such as medication administration.

#### Advanced EEG Techniques: Quantitative EEG Analysis

There is increasing interest in the application of mathematical transformations to raw EEG, termed quantitative EEG (QEEG), to enhance the efficiency of EEG interpretation, to allow for real-time bedside interpretation, and to identify subtle changes in brain activity that may not be evident on conventional intermittent review of raw waveforms by an electroencephalographer. The basis of QEEG analysis is transformation of raw time-domain (TD) EEG waveforms into a frequency-domain (FD) distribution using the fast Fourier transform (FFT); the FFT may be used to assess the relative contributions of various frequency bands of electrical brain activity to overall power (spectrograms) [147]. Amplitude integrated EEG (aEEG), in which the EEG signal from each hemisphere is filtered, rectified, and displayed as peak-to-peak amplitude on a compressed time scale, is routinely used at the bedside of high-risk critically ill neonates. aEEG has demonstrated correlations with encephalopathy severity and can be used to screen for seizures [148]. Additional analyses include assessment of rhythmicity, the degree of asymmetry between different regions, and the suppression ratio (defined as the percentage of EEG activity that falls below a set amplitude threshold).

Seizures typically manifest on FFT spectrograms as abrupt increases in power (“flames”) and rhythmicity [149]; QEEG has been used to augment cEEG review for seizure detection [150, 151]. QEEG has also demonstrated utility for the detection of ischemia related to vasospasm following subarachnoid hemorrhage [152, 153] or during carotid endarterectomy [154] in adults. Ischemia may manifest as decreased power in faster frequencies and increased power in slower frequencies, sometimes called the alpha/delta ratio [155]. Preliminary reports in both adult and pediatric populations suggest that changes in QEEG trends may precede other clinical signs in devastating but potentially reversible conditions, such as strokes or cerebral herniation events [156–158]. Despite the longstanding availability of QEEG, patterns of use are highly variable across centers and require further standardization [159].

### Optical Neuromonitoring

Optical neuromonitoring using near-infrared (NIR) light provides the unique ability for continuous noninvasive quantification of cerebral hemodynamic and metabolic risk factors for acute brain injury at the bedside [160, 161]. NIRS measurement of cerebral tissue oxygen saturation (StO_2_), physiologically analogous to regional tissue oxygen saturation and the tissue oxygenation index, is the most widespread optical technique [174]. StO_2_ assesses the balance of arterial oxygen delivery and tissue consumption and has repeatedly shown potential utility for rapid detection of cerebral hypoxia and ischemia. Compared to other modalities highlighted in this review, clinical NIRS devices are typically more portable and rapidly applied and do not require a clinical specialist. Ease of use has promoted increasing application in emergent and critical care settings [28, 162–180]. However, consensus on utility and standardized guidance for interpretation remains lacking [175, 178, 181–184]. Here we provide a technical overview of cerebral NIRS to elucidate current limitations and highlight emerging techniques and diagnostics with potential impact on the management of acute brain injury.

#### Technical Considerations

**Fig. 6:**
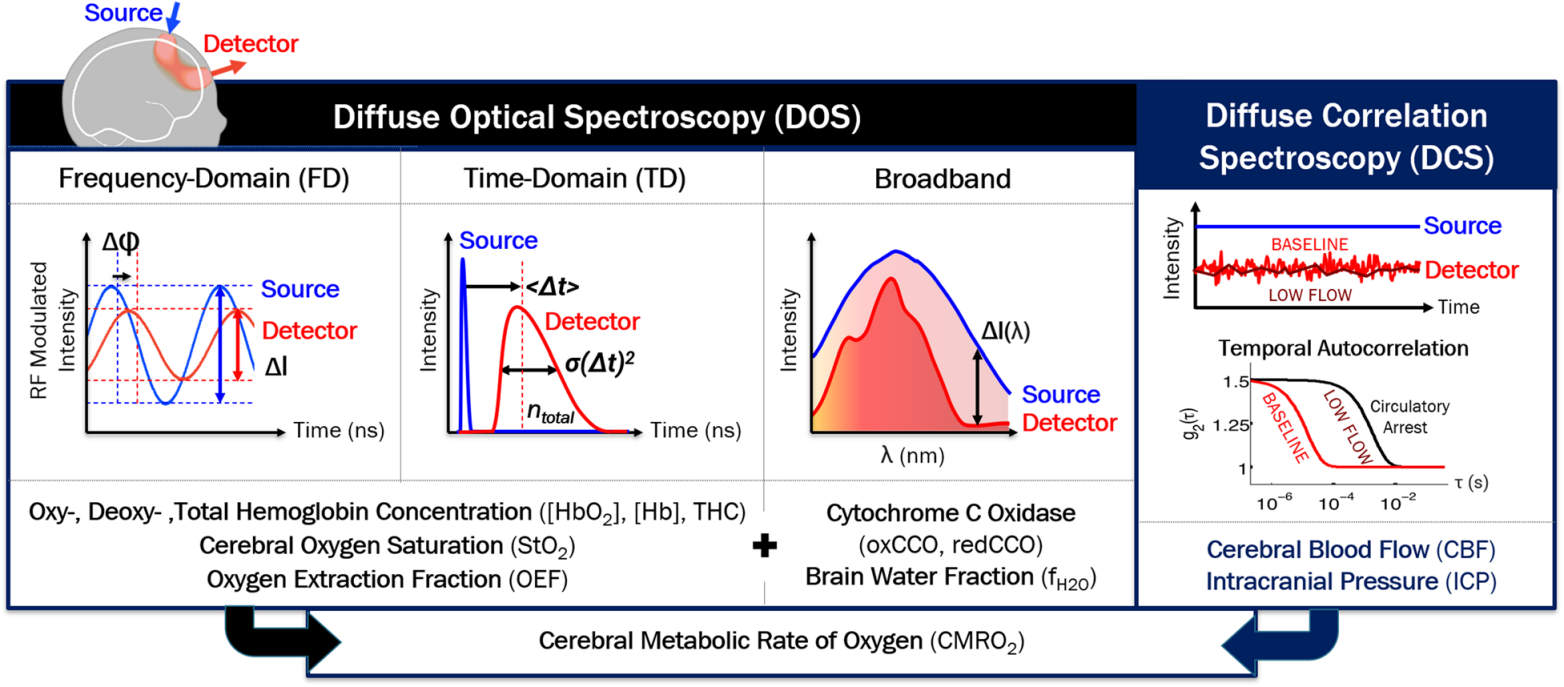
Advanced optical neuromonitoring techniques and quantified physiologic parameters. Near-infrared light travels noninvasively through tissue from a light source on the surface of the skin, to the brain, and back to a light detector in a “banana”-shaped distribution. In advanced near-infrared spectroscopy (NIRS), also known as diffuse optical spectroscopy (DOS), unique properties of the detected light permit quantification of the cerebral tissue concentration of numerous physiologically significant chromophores including oxyhemoglobin and deoxyhemoglobin, water and cytochrome c oxidase (CCO) [271]. In addition to changes in intensity (used by clinical NIRS), these properties include: φ, phase; I, intensity; < Δt > , mean arrival time of photons; *σ*(Δt)^2^, variance of arrival times; n_total_, total number of detected photons; and Δ*I*(λ), change in intensity across hundreds of wavelengths of NIR light. Diffuse correlation spectroscopy (DCS) is another near-infrared light technique that permits noninvasive measures of cerebral perfusion. The speed of red blood cell motion results in rapid light intensity fluctuations that may be characterized by empirically fitting for the decay of the temporal intensity autocorrelation curve (*g*_2_); the higher the blood flow, the more rapid fluctuations occur and the more rapid the decay of the *g*_2_ curve. This yields a blood flow index (BFI) which has been well-validated as a relative measure of cerebral blood flow. Techniques have also been developed to estimate intracranial pressure (ICP) from the pulsatile BFI waveform [251–254]

Within tissue, NIR photons are either scattered or absorbed; scattering events are roughly 100 times more likely than absorption events. This relative “transparency” of tissue permits photons, emitted from a light source on the surface of the skin, to travel to, and back from, the brain [161, 185]. The predominance of scattering allows accurate modeling of light transport as diffusing particles, each performing a “random walk,” through tissue from a light source to a light detector in a “banana-shaped” distribution pattern (Fig. 6). The detected light encodes physiologic information from this diffuse region of tissue as a function of the tissue’s absorption and scattering. Accurate quantification of absorption is required to spectroscopically resolve relative concentrations of oxyhemoglobin and deoxyhemoglobin and compute StO_2_. Current clinical pediatric NIRS devices use continuous-wave (CW) NIR light-emitting diode light sources, which enable compact, disposable patient interfaces but only permit signal contrast in detected light amplitude; population-based scattering assumptions are required to estimate absorption [186]. Advanced NIRS, also known as diffuse optical spectroscopy (DOS), provides improved quantitative StO_2_ accuracy using increased spectral and temporal information.

##### Advanced NIRS/DOS Techniques

In broadband or hyperspectral DOS, a CW white light source paired with a spectrometer light detector measures amplitude changes at hundreds of NIR wavelengths [187–189]. This increased spectral information provides the ability to estimate the contributions of tissue scattering, in addition to tissue chromophore absorption, to amplitude changes [190]. After accounting for tissue scattering, the high-resolution spectral absorption information permits  concentration determination of multiple tissue chromophores. In addition to oxyhemoglobin and deoxyhemoglobin, more weakly absorbing tissue chromophores with potential physiologic significance, including cytochrome c oxidase (CCO), water, and lipid, may also be quantified.

FD and TD DOS techniques provide the ability to quantify absolute values of tissue scattering and absorption properties [191–195]. In FD DOS, frequency-modulated light encodes amplitude as well as phase information. In TD DOS, an ultrafast pulse of light is attenuated and broadened as a function of absorption and scattering; time-gating of the detected pulse allows preferential selection of photons traveling deeper into tissue with greater brain sensitivity [196]. Multispectral FD and TD DOS measurements of the absorption coefficient enable absolute spectroscopic quantification of the oxyhemoglobin, deoxyhemoglobin, and total hemoglobin concentrations and more accurate calculation of StO_2_ based on these absolute values compared to CW NIRS. Total hemoglobin concentration provides an additional diagnostic measure of cerebral blood volume (CBV) [197].

In contrast to broadband DOS, specialized optoelectronics are typically required for each measurement wavelength, which increases the complexity and sampling time for multispectral measurements [198]. Due to cost and footprint limitations, 2–4 measurement wavelengths are common, which precludes quantification of chromophores other than oxyhemoglobin and deoxyhemoglobin. Development of hybrid broadband and FD and TD DOS approaches is ongoing to leverage their combined advantages [199–202].

##### Monitoring Perfusion

Diffuse correlation spectroscopy (DCS) and laser speckle contrast flowmetry are emerging optical neuromonitoring techniques that permit continuous noninvasive quantification of cerebral perfusion and remain robust in low-flow states. A cerebral blood flow index (BFI) (cm^2^/s) is derived from temporal and spatial characterization of rapid intensity fluctuations (termed “speckles”) resulting from the constructive and destructive interference of the scattering of coherent NIR light by moving red blood cells [160, 203–205]. In contrast to perfusion MRI and TCD, signal to noise is proportional to the amount of detected light and does not rely on intrinsic flow contrasts. Compared to DOS, quantified CBF contrasts provide improved sensitivity to the brain [206]. DCS measures of BFI have been repeatedly validated as an accurate surrogate of CBF in children and pediatric preclinical models [207, 208, 209–214] and provide sufficient temporal resolution to resolve blood flow pulsatility [215–218].

#### Emerging Clinical Applications

##### Neurometabolic Optical Monitoring

**Fig. 7:**
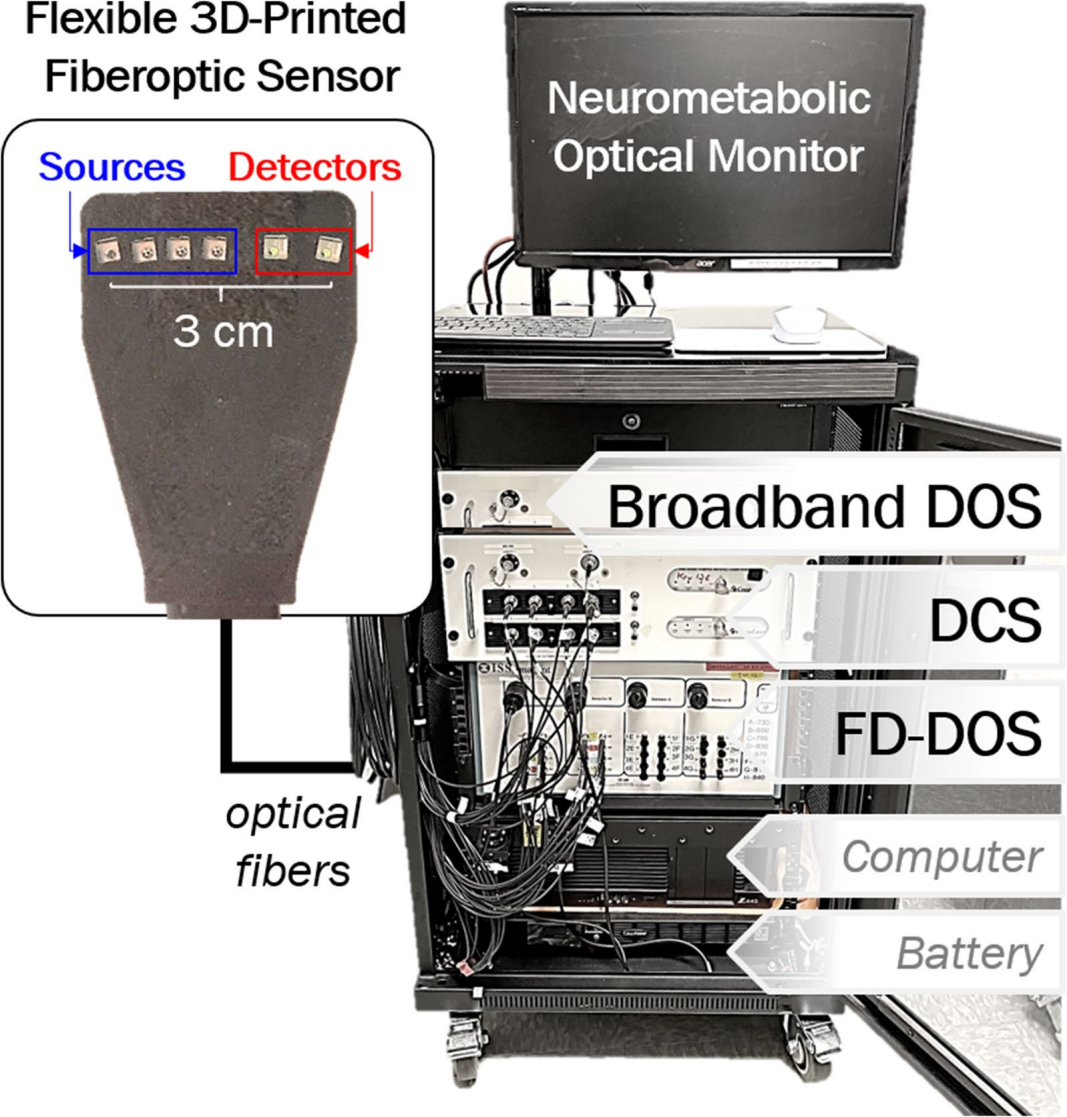
Advanced neurometabolic optical monitor. Pictured is a research-grade device, approved for investigational use only, integrating three advanced optical techniques: broadband diffuse optical spectroscopy (DOS), frequency-domain (FD) DOS, and diffuse correlation spectroscopy (DCS). These three techniques may be integrated within a single, compact and flexible, pediatric optical sensor pad shown in the top left

Several state-of-the-art diffuse optical neuromonitoring devices have implemented combined DCS and advanced DOS techniques for investigational use in pediatric settings [191, 219–229]. The advantages of this approach are two-fold. The accuracy of DCS BFI is significantly improved by absorption and scattering information measured by DOS [230]. Furthermore, concurrent monitoring of cerebral perfusion and oxygen saturation may be combined based on Fick’s principle to also monitor changes in the cerebral metabolic rate of oxygen in a single compact fiber optic sensor (Fig. 7) [231–233].

**Fig. 8:**
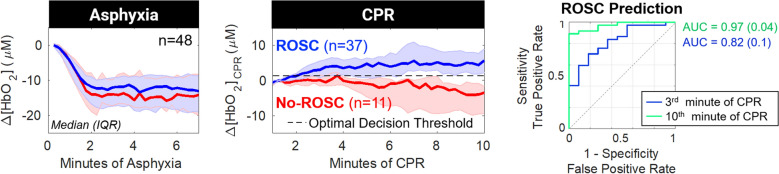
Changes in cerebral hemodynamics during asphyxia and cardiopulmonary resuscitation (CPR) in a pediatric swine model of asphyxia-associated cardiac arrest. The changes in cerebral oxy-hemoglobin concentration (Δ[HbO_2_]) from baseline during asphyxia (left), and from 1-min of CPR during CPR (center), are summarized and grouped by CPR outcome (achieved return of spontaneous circulation (ROSC), blue; No ROSC, red). Data are plotted as median (thick solid line) and interquartile range (IQR; thin solid line). The optimal, intra-arrest, decision threshold for ROSC is plotted (dotted line) for Δ[HbO_2_]_CPR_ (+ 1.3 µmol/L). The receiver operating characteristic curve for Δ[HbO_2_]_CPR_ prediction of ROSC has an area under the receiver operating characteristic curve (AUC) of 0.82 (0.1) in third minute of CPR and increases to 0.97 (0.04) in the tenth minute of CPR. (Adapted from Ko et al. [246] under Creative Commons CC BY license: http://creativecommons.org/licenses/by/4.0/.)

Longitudinal neurometabolic optical monitoring has elucidated risk factors for hypoxic–ischemic brain injury in several pediatric contexts, including during birth transition in healthy and high-risk neonates [220, 234–240], and during generalized seizures [241, 242], cardiac surgery requiring cardiopulmonary bypass [213, 225, 243, 244], and extracorporeal membrane oxygenation support [226, 245]. Quantification of oxygen delivery to the brain intra-arrest [246] (Fig. 8) and low-frequency blood flow oscillations post arrest [247] may provide novel noninvasive targets for physiologic optimization of resuscitation and postarrest care.

Broadband DOS quantification of CCO and water provides unique diagnostic utility and oxygen metabolism insights beyond the vascular compartment to intracellular mitochondria [189]. Temperature-dependent spectral features of water have been used in noninvasive brain temperature assessment [248]; water content may additionally provide diagnostic utility for monitoring of cerebral edema during extracorporeal support or following hypoxic–ischemic brain injury [249, 250].

##### ICP

**Fig. 9:**
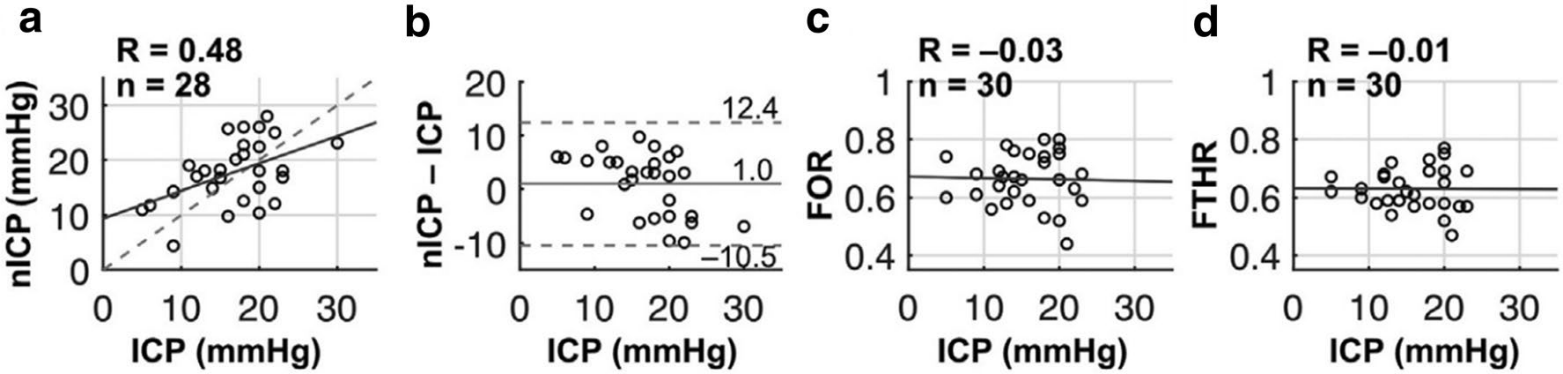
Validation of noninvasive intracranial pressure (nICP) versus invasive intracranial pressure (ICP) in neonates with hydrocephalus. **a** Intraoperative nICP was correlated with ICP acquired during CSF diversion surgery (solid line, linear best fit; dashed line, line of unity). **b**, Bland–Altman plot of the difference between nICP and ICP (solid line, mean difference; dashed lines, 95% limits of agreement, i.e., the mean ± 1.96 times the SD of the difference). **c**, **d**, Preoperative frontal-occipital horn ratio (FOR) (**c**) and frontotemporal horn ratio (FTHR) (**d**) as measured on brain ultrasound were not correlated with ICP (solid lines, linear best fit). (Adapted from Flanders et al. [228] with author permission under Creative Commons CC BY-NC-ND license: https://creativecommons.org/licenses/by-nc-nd/2.0/.)

Novel optical methods have recently demonstrated promising evidence of noninvasive ICP assessment [218, 251–254]. Initial optical approaches adapted TCD ultrasound techniques [255], relating pulsatility of arterial blood pressure versus CBF for the microvasculature, measured by DCS, to quantify critical closing pressure [251, 254]. TCD cross-validation demonstrated significant correlation and concordance across a wide range of critical closing pressures (up to 65 mm Hg) in healthy adults and adults with brain injury. This technique, termed noninvasive ICP (nICP) in Flanders et al., was found to significantly correlate with invasive manometer ICP measurements in 28 infants with emergent hydrocephalus, of whom 18 (64%) fulfilled criteria for intracranial hypertension (ICP ≥ 15 mm Hg) [228]. Critically, although nICP was able to discriminate infants with intracranial hypertension, clinical diagnostic ultrasound measures (frontal-occipital horn ratio and frontotemporal horn ratio) did not (Fig. 9).

A source of uncertainty in this initial optical approach is the necessity for arterial blood pressure pulsatility measurements, which were estimated by systolic and diastolic cuff measurements. Application of advanced machine learning models may enable ICP prediction based on standalone DCS BFI or FD DOS measurements of trends in oxyhemoglobin, deoxyhemoglobin, and total hemoglobin concentrations [252, 253, 256]. These results highlight the feasibility and potential utility for a point-of-care noninvasive optical assessment of ICP in emergent settings and as a continuous bedside monitor to improve detection and management of intracranial hypertension.

##### Cerebral Autoregulation

Noninvasive optical assessment of cerebral autoregulation via quantification of cerebrovascular pressure reactivity has emerged as a valuable diagnostic of acute brain injury vulnerability in children [29, 173, 175, 180, 257–259]. Optical approaches to quantify cerebral autoregulation in pediatric neurocritical care have commonly examined the correlation of spontaneous fluctuations in arterial blood pressure and clinical NIRS measures of CBV and StO_2_ as surrogates of CBF [257, 260–262]. An excellent review of current techniques and practical recommendations for data capture, with particular emphasis on neonatal monitoring, are summarized by Rhee et al. and Vesoulis et al. [180, 257]. The application of advanced optical techniques, including the use of DCS for direct assessment of alterations in CBF [226, 263–268], may improve diagnostic utility. Assessment of pressure passivity of cerebral metabolism in term HIE infants using broadband DOS measurement of changes in oxidized CCO demonstrated a significant association with MRI injury severity and neurodevelopmental outcomes at 1 year of age [239, 269].

## Discussion

Advancements in noninvasive quantification of cerebral physiology provide novel insights for improved detection and management of acute brain injury in children. Emerging MRI techniques uniquely afford whole-brain spatial characterization of cerebral perfusion, metabolism, and structural abnormalities for detection and quantification of injury. This is particularly critical for localized injuries due to trauma, stroke, or neoplasms. Timing of MRI following injury and dynamic changes in MR signals with age remain a challenge for interpretation and generalizability. HUS imaging has become another essential neurodiagnostic modality due to its accessibility, safety, and portability. The emergence of advanced ultrasound techniques, such as MVI, CEUS, and elastography, expand the quantitative and functional evaluation of many neurological pathologies. Continued optimization of acquisition parameters for efficacy and safety is underway. cEEG is critical for detection of dynamic background changes and seizures in critically ill children and neonates. QEEG is an evolving technology permitting real-time optimization of care in critically ill children. Optical neurometabolic monitoring of the brain is an emerging multiparameter modality that provides unique advantages in this respect but has limited utility in the detection of focal deep-brain injury where light is unable to penetrate. Use of cerebral NIRS for continuous StO_2_ neuromonitoring has been tempered by limitations in accuracy. Emerging advanced DOS and DCS techniques provide improved quantitative accuracy and novel physiologic information for injury detection. For both ultrasound and optical techniques, neurocritical care applications have largely been limited to neonates and infants; improving depth sensitivity is critical to characterizing focal deep-brain pathologies in older pediatric populations. Ongoing standardization of these emerging techniques will improve reproducibility and aid in necessary multicenter studies to establish high-quality evidence of clinical impact on neurodevelopmental outcomes.
