# Correction: Advanced Neuromonitoring Modalities on the Horizon: Detection and Management of Acute Brain Injury in Children

**DOI:** 10.1007/s12028-023-01743-z

**Published:** 2023-05-09

**Authors:** Tiffany S. Ko, Eva Catennacio, Samuel S. Shin, Joseph Stern, Shavonne L. Massey, Todd J. Kilbaugh, Misun Hwang

**Affiliations:** 1 Department of Anesthesiology and Critical Care, Children’s Hospital of Philadelphia, Philadelphia, USA; 2 Division of Neurology, Department of Pediatrics, Children’s Hospital of Philadelphia, Philadelphia, USA; 3 Department of Neurosurgery, Hospital of the University of Pennsylvania, Philadelphia, USA; 4 Department of Radiology, Children’s Hospital of Philadelphia, University of Pennsylvania, Philadelphia, USA

**Correction: Neurocrit Care** 10.1007/s12028-023-01690-9

In the original article, the phrase “electric field intensity temporal autocorrelation curve (*g*_2_)” has been corrected to “temporal intensity autocorrelation curve (*g*_2_)” in Fig. 6 caption.

